# Genital ulcer severity score and genital health quality of life in Behçet’s disease

**DOI:** 10.1186/s13023-015-0341-7

**Published:** 2015-09-22

**Authors:** Amal Senusi, Noha Seoudi, Lesley Ann Bergmeier, Farida Fortune

**Affiliations:** Centre for Clinical and Diagnostic Oral Sciences, Institute of Dentistry, Barts and the London School of Medicine and Dentistry, Queen Mary University of London, London, UK; Centre for Clinical and Diagnostic Oral Sciences, Institute of Dentistry, Barts and the London School of Medicine and Dentistry, Blizard Institute, 4 Newark Street, London, E1 2AT UK

**Keywords:** Behçet’s disease, Genital ulcer, Severity, Genital health quality of life

## Abstract

**Background:**

Behçet’s Disease (BD) is a chronic auto-inflammatory, multisystem relapsing/remitting disorder of unknown aetiology. Oro-genital ulceration is a key feature of the disease and has a major impact on the patients’ quality of life. Other clinical manifestations include ocular inflammation, rheumatologic and skin involvement, while CNS and vascular complications can lead to considerable morbidity. The availability of a valid monitoring tool for BD activity is crucial in evaluating the impact of the disease on daily life activity. The aims of this study were to validate a novel tool for monitoring genital ulceration severity in BD and to assess the impact of genital ulcers on the Genital Health Quality of Life (GHQoL).

**Methods:**

Genital Ulcer Severity Score (GUSS) was developed using six genital ulcer characteristics: number, size, duration, ulcer-free period, pain and site. A total of 207 BD patients were examined, (137 females: mean age ± SD: 39.83 ± 13.42 and 70 males: mean age ± SD: 39.98 ± 11.95) from the multidisciplinary Behçet’s Centre of Excellence at Barts Health NHS Trust. GUSS was used in conjunction with Behçet’s Disease Current Activity Form (BDCAF).

**Results:**

The over-all score of GUSS showed a strong correlation with all genital ulcer characteristics, and the strongest correlation was with the pain domain (r = 0.936; *P* < 0.0001). Ulcer average size and ulcer pain were the major predicting factors in GUSS (β = 0.284; β = 0.275) respectively, and P-values were significant. Multivariate regression analysis indicated that the ulcer pain, size and site are the main ulcer characteristics having an influence on the GHQoL (R^2^: 0.600; *P* < 0.0001).

**Conclusions:**

This study established the practicality of GUSS as a severity monitoring tool for BD genital ulcers and validated its use in 207 patients. Genital ulcers of BD have a considerable impact on the patients GHQoL.

**Electronic supplementary material:**

The online version of this article (doi:10.1186/s13023-015-0341-7) contains supplementary material, which is available to authorized users.

## Background

Behçet’s Disease (BD) is a chronic auto-inflammatory, multisystem, peri-vasculitis disorder [1, 2], first described by the Turkish dermatologist Hulusi Behçet in 1937 [3]. BD is characterised by recurrent mucocutaneous lesions [4]. Oro-genital ulcers are usually the first sign and the main classification criteria of BD patients [5]. Skin lesions, relapsing uveitis, and articular, neurologic, urologic, intestinal and pulmonary manifestations can cause serious disability and significant impairment in the quality of life [6]. The aetiology and pathogenesis of BD is not fully clarified, however, the BD symptoms are considered to be based on the correlation between intrinsic factors (genetic) and triggering extrinsic factors (microbial and/or environmental), hormonal and immune system dysregulation are implicated in causing both reversible and irreversible organ damage [7]. BD is diagnosed based on the clinical criteria as established by Mason and Barnes (1969), O’Duffy and Goldstein (1974) [8]. The international study group (ISG1990) criteria were subsequently published to include a positive Pathergy test as one of BD criteria [9]. The most recent diagnostic criteria is that of the International Team for the Revision of the International Criteria for BD (Davatchi et al. 2014), which used a numerical scoring system with a sensitivity of 98.2 % and a specificity of 95.6 % in a 27 country BD cohort [10, 11].

The prevalence of BD is highest in Middle Eastern countries such as Turkey where it occurs in approximately 370/100,000 inhabitants and in Iran with a prevalence of 80/100,000 [12]. BD also occurs in Central and far Eastern Asian countries and is said to track the “Old Silk Road” trading routes [13]. BD is less common in northern Europe and the USA: with 4.2/100,000 in Germany; 7.2/100,000 in France; 8.6/100,000 in the USA; and 0.64/100,000 in the United Kingdom respectively [12]. BD onset usually occurs in mid third to fourth decade of life with almost equal male to female ratio, although men often have more severe symptoms [14].

BD disease course, severity, and systemic involvement between patients is variable, and the treatment depends on gender, age and weight (for calculation of drug dose) at presentation, therefore, it is challenging to determine a single management strategy [15]. Colchicine is widely used in treatment protocols for the mucocutaneous manifestations of BD and corticosteroid therapies and immunomodulatory drugs including biologic drugs will control active disease and remission in cases of major organ involvement that are unresponsive to conventional therapy [16].

Genital ulcers caused by BD are the second most common manifestation of BD [17], occurring in 57 % to 96 % of patients [18–20]. However, Alekberova et al. [21] found that the two major diagnostic criteria, namely aphthous stomatitis and external genital ulcers, were found with the same frequency. Genital ulcers typically start as a tender nodule, becoming deep and painful: interfering with sitting, walking and causing dysfunction, and usually healing slowly with scarring [22]. In females they are typically found on the labia majora, labia minora, on the vulva, perineum, or perianal skin. In males ulcers are mostly seen on the scrotum, less frequently on the shaft of the penis and occasionally on the tip of the penis [12]. Genital ulceration in BD is not contagious and cannot be spread through sexual intercourse; however, bacterial colonisation of ulcers may be a risk factor for the transmission of infection to sexual partners. Genital ulcers in BD resemble oral ulcers in appearance and clinical course [9, 13] and have been classified into three groups: minor aphthae that are smaller than 1 cm, major aphthae larger than 1 cm, and herpetiform aphthae that are multiple, very small ulcers [23]. The complexity of systemic symptoms and resistance to conventional treatment of BD patients can lead to both a temporary and permanent functional disability while neurological involvement can lead to mental impairment [24, 25]. Previous studies indicate that mucocutaneous-symptoms may cause deterioration in personal relationships and daily activity and impact on the QoL of BD [26, 27].

Mucocutaneous lesions in BD are important in presentation and diagnosis, and are considered hallmarks of BD [28]. Monitoring approaches include patients’ medical history, physical examination and specific serological tests are mandatory, however, these methods have limitations in measuring the effect of the disease on organ function. A scoring system, such as GUSS, would be extremely useful in measuring BD severity and treatment efficacy in clinical trials and assessing disease status at a given time during cross sectional studies and in tracking the evolution of disease over time by longitudinal observation.

In this clinical prospective study, we have designed and validated a tool for measuring genital ulcerations that will benefit the assessment BD patients’ disease status with respect to current and prospective therapies. This study also adds to the previous data by analysing the impact of GUSS on daily activity. The four factors are; siting, walking, passing urine and sexual activity, that interfere with their GHQoL at the time of genital ulceration.

## Methods

**Fig. 1:**
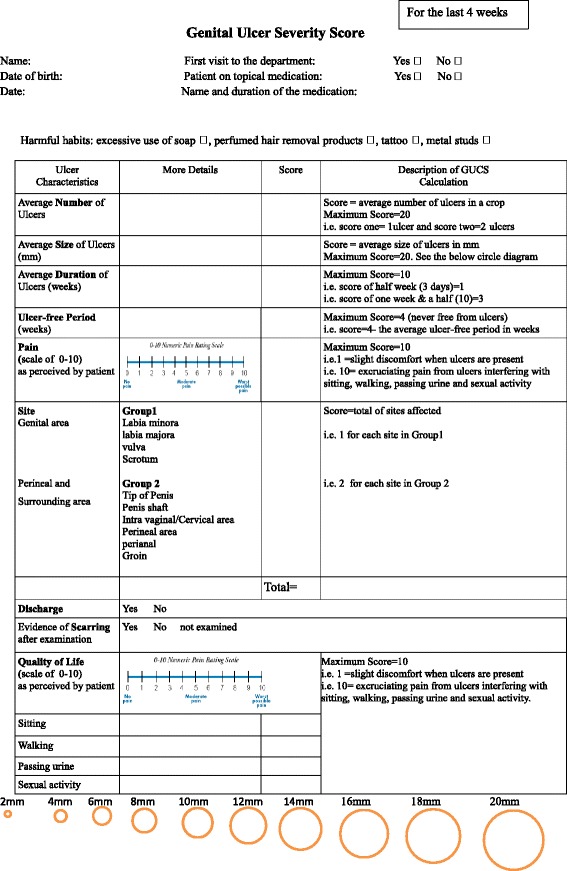
The Genital Ulcer Severity Score Form in BD

This prospective study is a part of The City Research Ethical Committee (COREC) approved study “Immune-regulation at the mucosal barrier” (P/03/122) and was carried out at Barts Health NHS Trust in full compliance with the Helsinki Declaration [29]. A cohort of 207 out of 263 fully consented BD patients classified according to the international study group (ISG) 1990 [30, 31], gave written consent for oral and genital assessment and examination. The GUSS forms (Fig. 1) were completed during the clinical assessment of the patients by the clinicians and senior specialist nurse at Behçet’s Centre of Excellence at Barts NHS Trust.

The exclusion criteria for the BD cohort were as follows; patients not fully diagnosed according to the ISG 1990; pregnant and lactating BD mothers were also excluded.

### Genital Ulcer Severity Score (GUSS) form

The GUSS form has been developed as a modification of the OUSS tool established by Tappuni et al. [32]. Six OUSS characteristics (number, size, duration, ulcer-free period, pain and site) were recorded and extended to include; evidence of scarring, and discharge to evaluate the severity of the genital ulceration in BD and to assess their effect on GHQoL at the time when the disease is active. Other confounding factors were also monitored such as potentially harmful habits including excessive use of soap, perfumed hair removal products, tattoos and metal studs in the genital area. Medications including the name, type, frequency and the duration of the therapy were also recorded for monitoring the efficacy of the treatment protocols for each patient.

To complete the GUSS form, the ulcers’ characteristics were converted into numerical values, in order to assess the genital ulcers in BD patients for the preceding 4 weeks.

The guidelines for GUSS domains calculation were as follows:

- **Average number of ulcers:** the score corresponds to the average number of ulcers per episode. i.e. Score 1 if there was one ulcer and score 2 if there were two ulcers etc. The maximum number is 20.
- **Average size of ulcers:** the score is the average of the ulcer diameter in millimetres. A size diagram was provided as circles at the bottom of the GUSS form. The maximum score is 20 mm. i.e. score 2 for the ulcer’s size of 2 mm etc. If the patient was uncertain of the size (between two different size circles) then an average of the ulcers’ size were recorded.
- **Duration of ulcers:** the score corresponds to the average duration of the ulcers and was calculated in ½ week units; i.e. Half week (3 days) = 1, one week and half = 3 etc. The maximum score is 10.
- **Ulcer-free period:** the score corresponds to the time free of ulcers in a period of 4 weeks; i.e. if the patient was free from ulcers for 1 week, the score = 3. The maximum score is 4 when the patient is never free from ulcers.
- **Pain:** The use of the validated pain visual analogue scale in the GUSS form allows the patients to quantify their pain during the time of ulceration. The minimum score is 0 (no pain) and the maximum score is 10 (severe pain).
- **Ulcer site:** this estimates the most frequent sites which are affected by ulcers in the genital skin/mucosa in males and females. Score 1 each for the most common affected sites and score 2 each for the less common affected sites in genital.
- **Discharge:** an evaluation by the patient answering if there was a fluid discharge or not at the time of ulceration.
- **Evidence of scarring:** evaluated by the clinician at the time of assessment.
- **Genital Health Quality of Life (GHQoL):** A scale from 0 (does not interfere with the GHQoL) to 10 (excruciating ulcers interfering with GHQoL) was included to correlate the GUSS with the patients’ GHQoL at the time of episodes in terms of (walking, sitting, passing urine and sexual activity).

### Behçet’s Disease Current Activity Form (BDCAF)

The BDCAF form [33] is a well-established tool for the assessment of BD activity in the clinic, which scores the history of clinical features; fatigue, headache, mouth ulcer and/or genital ulcer, skin lesions, joint involvement, blood vessel involvement, gastrointestinal and CNS complications, which present over the four weeks prior to the day of assessment. The form is completed by the patient in conjunction with a senior nurse (to help with any clarification required). The clinicians’ impression of disease activity was then included in the BDCAF score on scale from 0 to 12.

### Behçet’s disease treatment pathway

Treatment decisions varied, depending on the patients’ disease activity and symptoms and were based on the European League Against Rheumatism (EULAR) guidance for Behçet’s disease [34], and Behçet’s Centre of Excellence protocol for management of BD. These were followed to prevent/arrest any irreversible damages.

### Validation of GUSS form

The *validity* is defined as the degree to which a scale correlates with a theoretic concept [35]. To assess the validity of the GUSS: 1) we correlated the genital ulcer domains with the over-all GUSS, 2) The negative impact of genital ulceration on the patients’ GHQoL, therefore, the ulcer six characteristics were correlated with BDCAF and the GHQoL factors.

### Statistical analysis

The descriptive analysis was performed for mean and standard deviation values. The results were analysed by using the IBM SPSS Statistics software (version 20 for Windows; IBM Corporation, New York, NY, USA). Independent *t*-test and one-way ANOVA test were done to compare gender and age range groups with the GHQoL. The relationship between the variables was assessed by Pearson coefficient analysis. Multivariate regression analysis was performed for assessing the influence of the six characteristics of genital ulcers on the GUSS and the GHQoL, as the outcome measure resembled the normal distribution. The calculated *P* value < 0.05 was considered statistically significant. The correlation between the variables were ranked as “weak” or no association with values between 0 and 0.29 or (0 and −0.29), “moderate” with values between 0.3 and 0.69 or (−0.3 and −0.69), and “strong” if they were between 0.7 and 1 or (−0.7 and −1). The R-squared is the proportion of variance in the dependent variable that is explained by the additive combination of effects of the independent variables, and The ANOVA results indicate that the regression is significant or not significant. Beta value (standardised regression coefficients) is a measure of how strongly each predictor variables influences the dependent variable. The higher the beta value the greater the impact of the predictor variables on the dependent variable.

## Results

The genital ulcer severity of 137 females (mean age ± SD: 39.83 ± 13.42) and 70 males (mean age ± SD: 39.98 ± 11.95) was recorded. The frequency of genital ulceration in BD patients per a year was (mean of frequency ± SD: 7.13 ± 6.83), from the Multidisciplinary Behçet’s Centre of Excellence at Barts Health NHS Trust were monitored and calculated.

### Symptoms and Behçet’s disease activity

On the day of clinical assessment, all of BD patients were classified according to their BD activity, 61.4 % patients (*n* = 127) were active, whereas 33.8 % were inactive (*n* = 70). From the active disease group, a total of 69/207 (33.3 %) had oral ulceration, while (54/207, 26.1 %) had genital ulcers and most of the genital ulcers were herpetiform and minor in size. Almost 26 % of patients had a fluid discharge during the ulceration period, and 18 % had scarring in their genital area. In term of harmful habits, 2 patients had a tattoo, 1 patient had metal studs and 5 patients was used excessive soap to clean the genital area. Only 19/207 (9.18 %) patients had oral and genital ulceration at the same time. 21.7 % (45/207) of patients presented with skin manifestations such as erythema nodosum, pseudofolliculitis and papulopustular lesions, while joint and central nervous system manifestations, commonly presented as headache, were 73/207 (35.2 %) and 42/207 (20.2 %) respectively. Ocular manifestations occurred in 33/207 (15.9 %) patients (Table 1).

**Table 1:** Behçet's disease clinical systemic activity^a^

BD clinical systemic activity^a^	Pt. Number/total	%
Mouth	69/207	33.3 %
Genital	54/207	26.1 %
Mouth and Genital	19/207	9.18 %
Eyes	33/207	15.9 %
Skin	45/207	21.7 %
Joints	73/207	35.2 %
CNS	42/207	20.2 %
Active BD	127/207	61.4 %
Inactive BD	70/207	33.8 %

^a^BD activity on the day of clinical assessment

### Treatment modalities

About 20.3 % (42/207) were treated by multiple (more than 2) immunomodulatory medicines, 30 % (62/207) patients received two immunomodulatory medications. 51 received one immunomodulatory medication and 24 patients out of 207 required no medication at the time of presentation.

The most common immunomodulatory medications were as follows: Prednisolone 41 % (85/207) used predominantly during relapsed episodes; Colchicine, 39.6 % (82/207); Azathioprine, 30.4 % (63/207); Mycophenolate Mofetil (MMF) 9.6 % (20/207) and Methotrexate, 3.4 % (7/207). Infliximab was prescribed to 7.2 % (15/207); Humira 5.7 %. Topical corticosteroids for the oral ulcers were used in 58.5 % (121/207) of patients who used either Betamethasone mouthwash or Triple Therapy Mouthwash (1 tablet Betamethasone 500 mcg + 1 tablet Doxycycline 100 mg + 1 ml Nystatin 100.000 unit/ml dissolved in 10 ml of water). The patients are required to keep this solution in their mouth for 3 min and no food is to be taken for 1 h after use. These were prescribed for the patients with high OUSS score for use up to 4 times a day. 17.4 % (36/207) of patients used topical steroids for genital ulcers. However, only 5 patients were using the topical steroid medication for their skin manifestations (Table 2).

**Table 2:** Medications used by our BD cohort

BD medication	Pt. Number/total	%
No BD medication	24/207	11.6 %
One systemic medicine	51/207	24.6 %
Two systemic medicines	62/207	30 %
More than two systemic medicines	42/207	20.3 %
Medications’ name		
Prednisolone	85/207	41 %
Colchicine	82/207	39.6 %
Azathioprine	63/207	30.4 %
Mycophenolate Mofetil (MMF)	20/207	9.6 %
Infliximab	15/207	7.2 %
Humira	12/207	5.7 %
Methotrexate	7/207	3.4 %
Topical steroids oral medicine	121/207	58.5 %
Topical steroids genital medicine	36/207	17.4 %
Topical steroids skin medicine	5/207	2.41 %

### The site of genital ulceration and its effect on the GHQoL

**Fig. 2:**
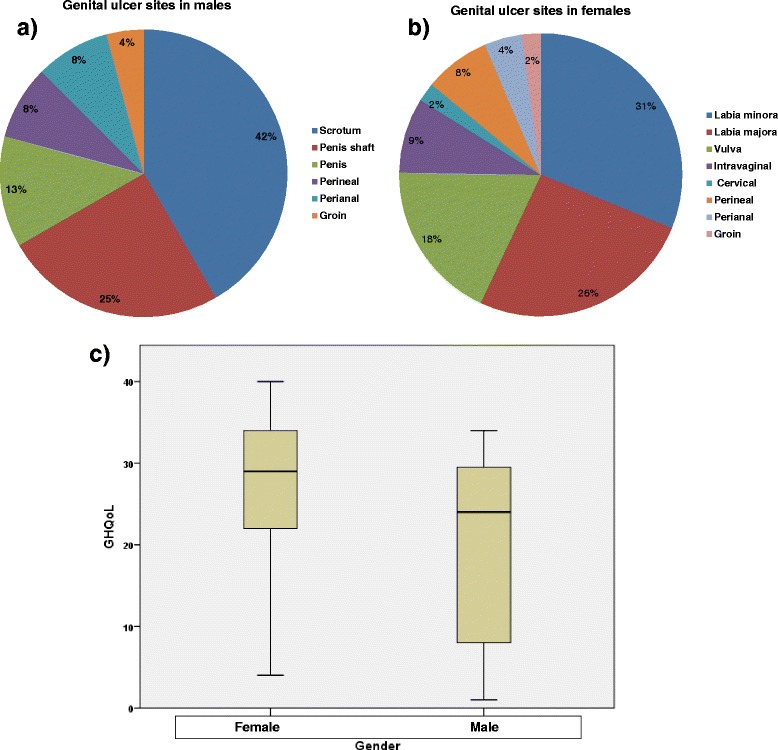
Genital ulcers in males and females. **a** Genital ulcer distribution in males, **b** Genital ulcer distribution in females **c** GHQoL and gender

The genital ulcers in males were most common in the scrotum (42 %, *n* = 10) followed by the penis shaft (25 %, *n* = 6) and the tip of the penis (13 %, *n* = 3). Ulcers in perianal and perineal area in males occurred in 8 %, (*n* = 2) each. In females the genital ulcers were most frequently recorded in the labia minora (31 %, *n* = 29), followed by the labia majora at (26 %, *n* = 24). Vulvar ulcers occurred in 18 % of patients (*n* = 17) while only 9 % presented with intravaginal ulcers. Perineal and perianal ulcers were recorded in 8 % and 4 % respectively, while ulcers in the cervical and groin area were rare (Fig. 2a, b). Statistically, the result illustrates that male and female groups are not equal in their GHQoL at the time of the genital ulceration (*p* = 0.027). Women had worse GHQoL than men. However, due to the unequal number of male and female participants, this finding cannot be substantiated in this study (Fig. 2c).

### The effect of age on GUSS and GHQoL

**Fig. 3:**
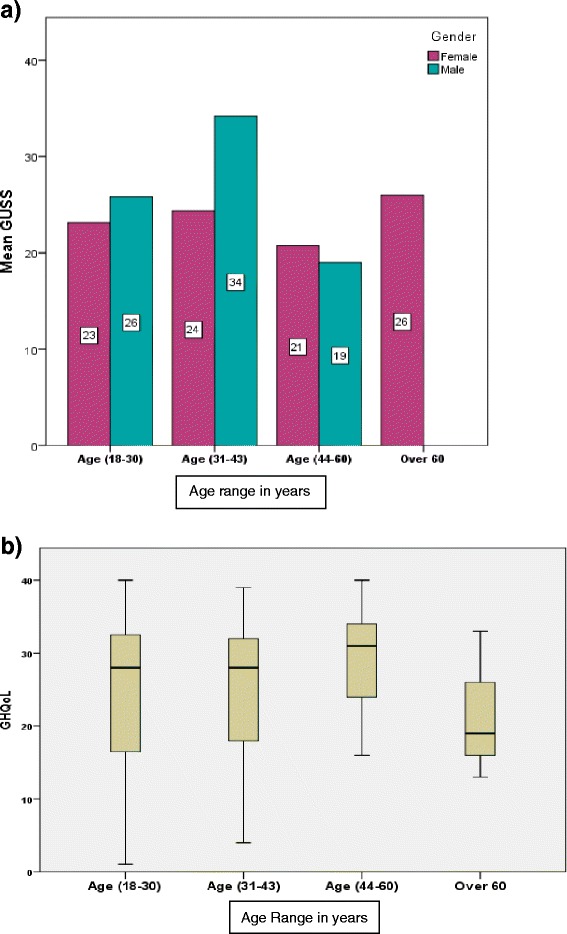
GUSS and Genital Health Quality of Life with BD patients’ age. **a** GUSS and the age **b** GHQoL and age range

Genital ulcers were widely distributed with a high severity score in patients between age ranges (18–30) and (31–43), within these age ranges males were higher in their GUSS than females (Fig. 3a), this was consistent with the BD literature [36, 37]. GUSS mean declined in the higher age range (44–60) in both genders with no significant difference between males and females. Interestingly high severity in GUSS was seen in women over 60 years of age (but not men). However, there is no difference in the GHQoL status between our male and female cohorts in all age ranges (*P* = 0.345) was shown in (Fig. 3b).

### Assessment of GUSS form

The correlation coefficient between genital ulcer characteristics and over-all GUSS showed a strong positive correlation and P values were significant with all ulcer domains (Table 3).

**Table 3:** Correlation of GUSS with ulcer characteristics

Correlations								
		GUSS	Ulcer Average Number	Ulcer Average Size (mm)	Ulcer Duration (weeks)	Ulcer Free-Period (weeks)	Ulcer Pain	Ulcer Site
Ulcer Average Number	Pearson Correlation	.836	1.000					
	Sig. (1-tailed)	.000*						
Ulcer Average Size (mm)	Pearson Correlation	.868	.572	1.000				
	Sig. (1-tailed)	.000*	.000*					
Ulcer Duration (weeks)	Pearson Correlation	.873	.712	.662	1.000			
	Sig. (1-tailed)	.000*	.000*	.000*				
Ulcer Free-Period (weeks)	Pearson Correlation	.855	.646	.679	.830	1.000		
	Sig. (1-tailed)	.000*	.000*	.000*	.000*			
Ulcer Pain	Pearson Correlation	.936	.767	.788	.746	.747	1.000	
	Sig. (1-tailed)	.000*	.000*	.000*	.000*	.000*		
Ulcer Site	Pearson Correlation	.861	.809	.657	.721	.742	.775	1.000
	Sig. (1-tailed)	.000*	.000*	.000*	.000*	.000*	.000*	

Significant values are indicated in bold

*P-value < 0.0001 (SPSS)

The strength of the correlation and the influence of the variables on GUSS are explained by (R^2^), and in this case the value was 0.998, this suggests that our model (which includes the six ulcer characteristics) explains 99.8 % of the over-all GUSS, and the regression analysis of GUSS with the six ulcer characteristics is statistically significant (*P* < 0.0001).

Beta value results showed that average ulcer size and then ulcer pain were the major predictive factors in GUSS (Tables 4 and 5).

**Table 4:** Model summary of GUSS with ulcer characteristics

		GUSS
Model Summary	R	.999^a^
	R Square	.998
	Sig. (ANOVA)	.000*

^a^Predictors: (Constant), Ulcer Site, Ulcer Average Size (mm), Ulcer Duration (weeks), Ulcer Average Number, Ulcer Free-Period (weeks), Ulcer Pain

Significant values are indicated in bold

*P- value < 0.0001 (SPSS)

**Table 5:** Multivariate regression analysis of ulcer characteristics with GUSS in BD

Coefficients*		
	Standardized Coefficients	Sig.
	Beta	
	GUSS	
Ulcer Average Number	.172	.ooo*
Ulcer Average Size (mm)	**.284**	.ooo*
Ulcer Duration (weeks)	.193	.ooo*
Ulcer Free-Period (weeks)	.111	.ooo*
Ulcer Pain	**.275**	.ooo*
Ulcer Site	.100	.ooo*

^a^Dependent variable: Genital Ulcer Severity Score (GUSS)

Significant values are indicated in bold

*P- value < 0.0001 (SPSS)

### The effect of genital ulcer characteristics on the BDCAF and GHQoL

The correlation between genital ulcer domains and the total of GHQoL factors; sitting, walking, passing urine, and sexual activity, using the Pearson coefficient, demonstrated a positive moderate correlation with the pain domain (r: 0.660; *P* < 0.0001), with ulcer average size (r: 0.447; *P* < 0.0001), and ulcer site (r: 0.383; *P* = 0.003). The relationship between genital ulcer domains and BDCAF showed a positive moderate correlation; with ulcer duration (r: 0.375; *P* = 0.003), then with ulcer average number (r: 0.368; *P* = 0.004) and have positive weak correlations with the rest of the ulcer characteristics (Table 6).

**Table 6:** Correlation of GHQoL factors, BDCAF with ulcer characteristics

Correlations							
		Sitting	Walking	Passing Urine	Sexual Activity	GHQoL	BDCAF
Ulcer Avenge Number	Pearson Correlation	.276	.120	.331	.123	252	.363
	Sig. (1-tailed)	.024*	198	.008*	.192	.035*	.004*
Ulcer Average Size (mm)	Pearson Correlation	.367	.456	.419	.236	.447	.107
	Sig. (I-tailed)	.004*	.000*	.001*	.046*	.000*	.225
Ulcer Duration (weeks)	Pearson Correlation	-.024	.119	-.021	-.265	-.113	.375
	Sig. (1-tailed)	.434	.201	.440	.029*	.203	.003*
Ulcer Free-Period (weeks)	Pearson Correlation	.247	.139	.152	.145	.200	.250
	Sig. (1-tailed)	.039*	.163	.141	.153	.078	.037*
Ulcer Pain	Pearson Correlation	.640	.654	.494	.445	.660	.189
	Sig. (1-tailed)	.000*	.000*	.000*	.000*	.000*	.089
Ulcer Site	Pearson Correlation	.376	.419	.320	.213	.383	.218
	Sig. (1-tailed)	.003*	.001*	.001*	.065	.003*	.060

Significant values are indicated in bold

*P- value <0.05 (SPSS)

The multivariate linear regression analysis (Tables 7 and 8), indicated that ulcer characteristics had an influence on the total of GHQoL (R^2^: 0.600; *P* < 0.0001). The beta values indicate that most of the contributions to difficulties in sexual activity, walking and sitting were due to ulcer pain and duration of ulceration, respectively. Ulcer pain was the only factor making a statistical significant contribution to passing urine.

**Table 7:** Model summary of GHQoL factors and BDCAF with ulcer characteristics

		Sitting	Walking	Passing Urine	Sexual Activity	GHQoL	BDCAF
Model Summary	R	.724^a^	.783^a^	.624^a^	.615^a^	.775^a^	.504^a^
	R Square	.524	.614	.389	.378	.600	.254
	Sig. (ANOVA)	.000*	.000*	.001*	.001*	.000*	.033*

^a^Predictors: (Constant). Ulcer Site. Ulcer Average Size (mm). Ulcer Duration (weeks). Ulcer Average Number. Ulcer Free-Period (weeks). Ulcer Pain

Significant values are indicated in bold

*P- value < 0.05 (SPSS)

**Table 8:** Multivariate regression analysis of ulcer characteristics with GHQoL factors in BD

Coefficients^a^								
	Standardized Coefficients	Sig.	Standardized Coefficients	Sig.	Standardized Coefficients	Sig.	Standardized Coefficients	Sig.
	Beta		Beta		Beta		Beta	
	Sitting		Walking		Passing urine		Sexual activity	
Ulcer Average Number	.105	.405	−.121	.287	.248	.087	.041	.773
Ulcer Average Size (mm)	.077	.514	.180	.092	.231	.086	−.007	.960
Ulcer Duration (weeks)	−.226	.047*	−.275	.008*	−.161	.204	−.434	.001*
Ulcer Free-Period (weeks)	.160	.154	.093	.355	.069	.583	.160	.211
Ulcer Pain	.571	.000*	.554	.000*	.368	.010*	.485	.001*
Ulcer Site	.152	.238	.316	.008*	.045	.757	.088	.548

^a^Dependent Variables: Sitting, Walking, Passing urine and Sexual activity

Significant values are indicated in bold

*P- value < 0.05 (SPSS)

## Discussion

The present study is the first to use GUSS to assess genital ulcer severity, monitor disease progression and evaluate the impact of genital ulceration on Genital Health Quality of Life (GHQoL) in BD.

QoL is a multidimensional measurement relating to all areas of human behaviour, which has been difficult to define and to measure because cultural, ethnic, religious and personal values influence the way that an individual responds to changes in QoL. Health Quality of Life (HQoL) attempts to measure how disease affects the quality of life. [38].

BD is a chronic inflammatory multisystem disease with periods of exacerbation and remission that negatively impacts on the patients’ QoL, both due to disease itself or the impact of its symptoms [39].

Our results demonstrate that genital ulcers are common in females and can occur on the labia, vulva and intravaginally. They present most commonly on the labia minora followed by labia majora. Genital ulcers are less common in males and are found most frequently on the scrotum. The frequent occurrence of genital ulcers in adults between 20 to 40 years may be related to a combination of environmental and hormonal factors [5]. Most of the genital ulcers in females were herpetiform in morphology, although minor and major aphthous ulcers also occur. The over-all GUSS score being higher in patients with major and herpetiform ulcers. Our results contribute to previous studies which indicate that BD severity may lessen as the age of the patient increases [40, 41]. GHQoL in females was worse when compared to the male group. This may be the result of the complexity of the anatomical structures and thin mucocutaneous tissue in women compared to men.

The multivariate regression analysis demonstrates a strong positive correlation between genital ulcer characteristics and the over-all GUSS as well as indicating that the ulcer average size and ulcer pain were major predictive factors on over-all GUSS.

The pain score significantly correlated with the average ulcer size particularly in patients with the herpetiform ulceration. This is an important observation as these ulcers are frequently missed on examination by clinicians, leaving patients unable to carry out basic activities such as sitting, walking, passing urine and sexual intercourse during periods of genital ulceration.

Not surprisingly, the correlation between genital ulcer characteristics and BDCAF was moderate since not all patients had active systemic disease and genital ulcers concomitantly. A finding is supported by previous clinical observation and the BD epidemiology literature.

In patients with active genital ulcers, the pain, size and site are the main ulcer characteristics which correlated with GHQoL [see Additional file 1].

Sexual activity was one of the major factors affecting the GHQoL of BD patients which may be linked to 1) constant pain before and after sexual intercourse, 2) the expectation of developing ulcers subsequent to sexual intercourse and 3) the sexual partner may have a large psychological component.

Psychological support is needed in patients with genital ulceration in BD. The Behçet’s Centre of Excellence at Barts NHS Trust provides such support.

However, to date there is little information in the literature regarding the negative impact of BD on the quality of patients’ sexual life [42]. This suggests an unmet need to evaluate the impact of genital ulceration on the quality of life of BD patients, which might provide insights into the requirements of the patients and lead to a step change in the treatment and support offered for this very sensitive area of health care and highlights the need and value of a multidisciplinary health team to provide appropriate medical and psychological care for patients with chronic diseases.

## Conclusion

The initial estimation of GUSS validity suggested that this instrument is a practicable and valid tool for assessing disease activity, disease progression and GHQoL.

Further effectiveness of this scoring system will become more apparent over the next few years as the use of GUSS has now become an integral part of our routine patient clinical practice.

## Additional file

Additional file 1:
**GUSS, BDCAF and GHQoL differences based on patients’ gender (Graph 1) and age range (Graph 2).** Sexual activity in both genders was the highest factor of GHQoL negatively affected by GUSS. BDCAF was very similar in males and females. GUSS and BDCAF declined with age. GHQoL became worse with age. However, in a group of patients’ age over 60, the GHQoL improved and was reflected in a decrease in GUSS score. (PDF 85 kb)

## Abbreviations

BD: Behçet’s disease
BDCAF: Behçet’s disease current activity form
GHQoL: Genital health quality of life
GUSS: Genital ulcer severity score
ISG: International study group
OUSS: Oral ulcer severity score
