# Supplementary material for: Genital ulcer severity score and genital health quality of life in Behçet’s disease
Source: Orphanet J Rare Dis. 2015 Sep 22;10:117. doi: 10.1186/s13023-015-0341-7 (PMC4579635; doi:10.1186/s13023-015-0341-7)

(Graph 1), GUSS, BDCAF and GHQoL differences based on gender

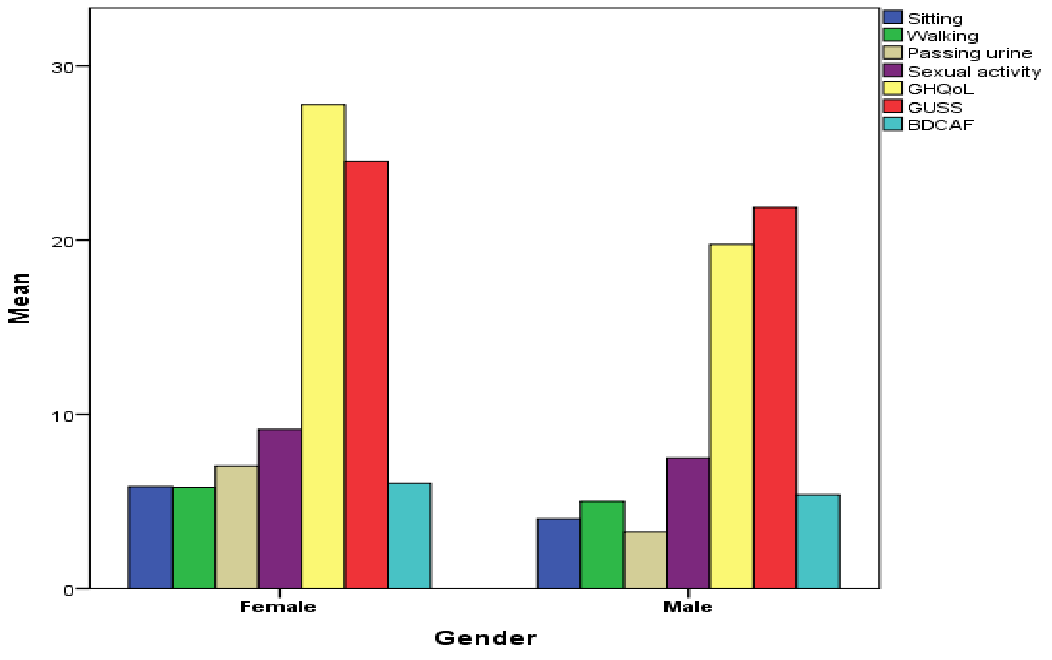

(Graph 2), GUSS, BDCAF and GHQoL differences based on patients' age range

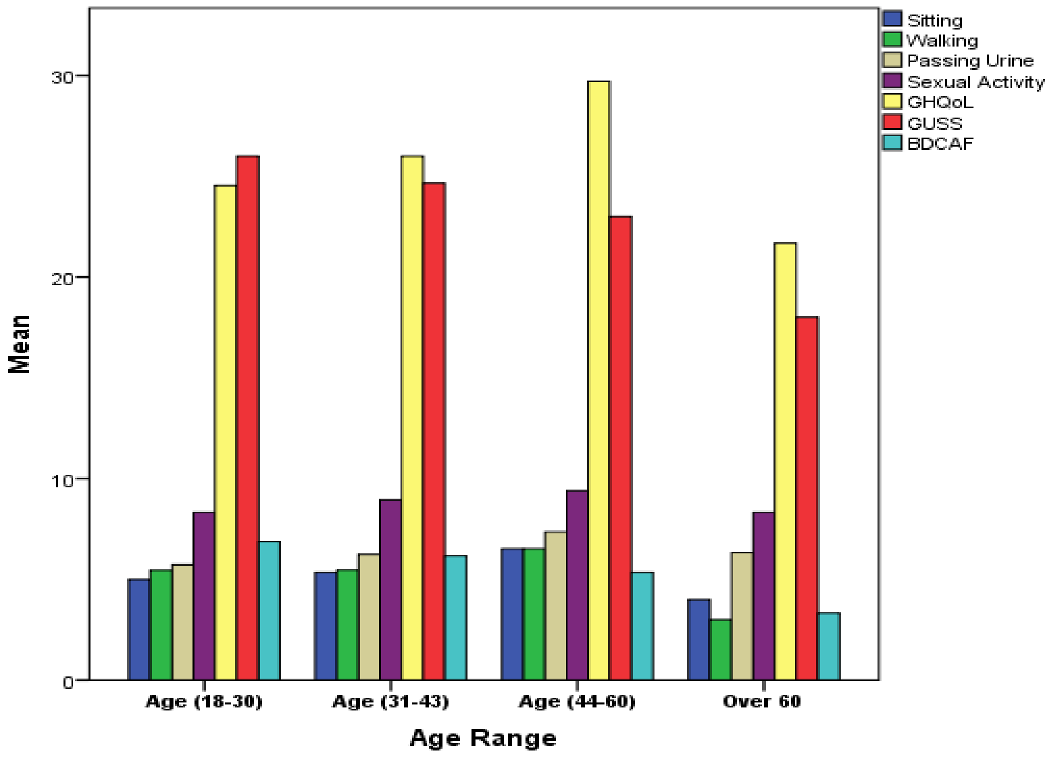

Supplement: Additional file 1: — GUSS, BDCAF and GHQoL differences based on patients’ gender (Graph 1) and age range (Graph 2). Sexual activity in both genders was the highest factor of GHQoL negatively affected by GUSS. BDCAF was very similar in males and females. GUSS and BDCAF declined with age. GHQoL became worse with age. However, in a group of patients’ age over 60, the GHQoL improved and was reflected in a decrease in GUSS score. (PDF 85 kb) [file 13023_2015_341_MOESM1_ESM.pdf]
